# Retraction: Smoothened regulates migration of fibroblast-like synoviocytes in rheumatoid arthritis *via* activation of Rho GTPase signaling

**DOI:** 10.3389/fimmu.2026.1937218

**Published:** 2026-07-21

**Authors:** Frontiers Editorial Office

The journal retracts the 15 September 2017 article cited above.

“Following publication, concerns were raised regarding the integrity of the images in the published figures. An investigation was conducted in accordance with Frontiers’ policies. It was concluded that the findings and conclusions of the article were unreliable, and the article has been retracted.

This retraction was approved by the Chief Editors of Frontiers in Immunology and the Chief Executive Editor of Frontiers. The authors were informed of this retraction.”

